# Mitochondrial group I and group II introns in the sponge orders Agelasida and Axinellida

**DOI:** 10.1186/s12862-015-0556-1

**Published:** 2015-12-12

**Authors:** Dorothée Huchon, Amir Szitenberg, Sigal Shefer, Micha Ilan, Tamar Feldstein

**Affiliations:** Department of Zoology, George S. Wise Faculty of Life Sciences, Tel Aviv University, Tel Aviv, 6997801 Israel; The Steinhardt Museum of Natural History, Israel National Center for Biodiversity Studies, Tel Aviv University, Tel Aviv, 6997801 Israel; Current address: School of Biological, Biomedical and Environmental Sciences, University of Hull, Hull, HU6 7RX UK

**Keywords:** Porifera, *Agelas*, *Axinella*, *Cymbaxinella*^*p*^, Horizontal gene transfer, *cox1*, Mitochondria, Group I intron, Group II intron

## Abstract

**Background:**

Self-splicing introns are present in the mitochondria of members of most eukaryotic lineages. They are divided into Group I and Group II introns, according to their secondary structure and splicing mechanism. Being rare in animals, self-splicing introns were only described in a few sponges, cnidarians, placozoans and one annelid species. In sponges, three types of mitochondrial Group I introns were previously described in two demosponge families (Tetillidae, and Aplysinellidae) and in the homoscleromorph family Plakinidae. These three introns differ in their insertion site, secondary structure and in the sequence of the LAGLIDADG gene they encode. Notably, no group II introns have been previously described in sponges.

**Results:**

We report here the presence of mitochondrial introns in the cytochrome oxidase subunit 1 (COI) gene of three additional sponge species from three different families: *Agelas oroides* (Agelasidae, Agelasida)*, Cymbaxinella*^*p*^*verrucosa* (Hymerhabdiidae, Agelasida) and *Axinella polypoides* (Axinellidae, Axinellida). We show, for the first time, that sponges can also harbour Group II introns in their COI gene, whose presence in animals’ mitochondria has so far been described in only two phyla, Placozoa and Annelida. Surprisingly, two different Group II introns were discovered in the COI gene of *C. verrucosa.* Phylogenetic analysis indicates that the Group II introns present in *C. verrucosa* are related to red algae (Rhodophyta) introns.

**Conclusions:**

The differences found among intron secondary structures and the phylogenetic inferences support the hypothesis that the introns originated from independent horizontal gene transfer events. Our results thus suggest that self-splicing introns are more diverse in the mitochondrial genome of sponges than previously anticipated.

**Electronic supplementary material:**

The online version of this article (doi:10.1186/s12862-015-0556-1) contains supplementary material, which is available to authorized users.

## Background

Mitochondrial introns are mobile genetic elements that form self-splicing RNA molecules. They are divided into Group I and Group II introns depending on their secondary structure and splicing mechanism [1]. Unlike spliceosomal introns, also called Group III introns, Group I and II introns encode other protein-coding genes in one of their loop regions. These protein-coding genes can be mitochondrial genes involved in the oxidative phosphorylation pathway [2] or, more frequently, enzymes, which catalyse their mobility. Most Group I introns encode homing endonuclease genes (HEG) and/or maturase of the LAGIDADG family, while most Group II introns encode a reverse transcriptase (RT) (review in [3–5]). Group I introns are present in the nuclear and organellar genomes of all three domains of life, while Group II introns are only present in prokaryotes and organellar genomes [3–5]. Within mitochondrial genomes, Group I introns are preponderant in fungi, Group II introns are predominant in plants [5], and both groups are rare in Metazoa. However, it should be noted that some eukaryotic groups are still poorly represented in mitochondrial databases. Group I introns have been found in Placozoa [6, 7], Anthozoa (corals, soft corals, sea anemones) (e.g., [2, 8–11]) and Porifera (sponges) [12–16], while Group II introns have been found in Placozoa [6, 7] and Annelida (a catworm of the genus *Nephtys*) [17]. Within these phyla, they are sporadically encountered in unrelated families. This patchy distribution and the fact that the intron phylogeny does not fit the species’ phylogeny support the view that they can be horizontally transmitted [10–13, 15, 18].

In sponges, mitochondrial introns have been found to be present in three species of the Plakinidae family (Homoscleromorpha) [14], in four species of the Tetillidae family (Demospongiae) [13] and in a member of the Aplysinidae family (Demospongiae) [15]. All sponge introns belong to Group I and were found to be inserted in the cytochrome oxidase subunit 1 (COI) gene. Interestingly, three different forms of introns were found, differing in their insertion site and secondary structure. These introns have been called intron 714, intron 723 and intron 870, based on their insertion sites [13]. The sponge COI sequences usually harbour only a single intron, except for *Plakinastrella onkodes* [14], which contains both intron 714 and intron 723. All sponge introns were found to encode a HEG from the LAGLIDADG family, except for intron 714 of *P. onkodes*, which does not encode any ORF [14]. Interestingly, the LAGLIDADG sequences were found to be divided into three unrelated clades depending on their insertion site [14].

We show here the presence of Group I mitochondrial introns in the COI gene of three additional Demosponge species: 1- *Agelas oroides* (Schmidt, 1864)*;* 2- *Axinella polypoides* Schmidt, 1862; and 3- *Axinella verrucosa* (Esper, 1794). It is worth noting that the genus *Axinella* is not monophyletic. Indeed the two *Axinella* species belong to different families, Axinellidae (*A. polypoides*) and Hymerhabdiidae (*A. verrucosa*), while *A. oroides* belongs to Agelasidae [19–21]. To avoid confusion, the PhyloCode nomenclature of Gazave et al. [19] will be used in this manuscript. The name *Cymbaxinella*^*p*^*verrucosa* is thus used to identify *A. verrucosa.* We also describe, for the first time, the presence of Group II introns in sponges. Indeed, the sponge *C. verrucosa* was found to harbour in its COI gene two Group II introns in addition to one Group I intron*.* Our results suggest that self-splicing introns are more diverse in the mitochondrial genome of sponges than previously anticipated.

## Results

### Sequences characteristics

As part of a survey of the Israeli sponge fauna, a ~1,200 bp fragment of the COI gene [12] was amplified from 42 specimens belonging to 30 different species (Additional file 1). Surprisingly, the PCR led to products longer than expected in three species: *A. oroides* (2,755 bp)*, A. polypoides* (3,032 bp) and *C. verrucosa* (7,188 bp). The products were sequenced using Sanger sequencing (see Methods). Sequence alignment of the sequenced fragments, together with sequences from closely-related sponge species, revealed the presence of several introns in the sequenced fragments. Two introns, introns 723 and 870, were found to be present in both *A. oroides* and *A. polypoides* sequences. Three introns, introns 723, 966 and 1141, were identified in *C. verrucosa.* To confirm that the obtained sequences were not a result of contamination, one additional *A. oroides*, one additional *A. polypoides*, and two additional *C. verrucosa* COI fragments were sequenced. The sequence of these additional specimens was found to be identical to the original sequences, supporting the view that these samples are not contaminations.

**Fig. 1:**
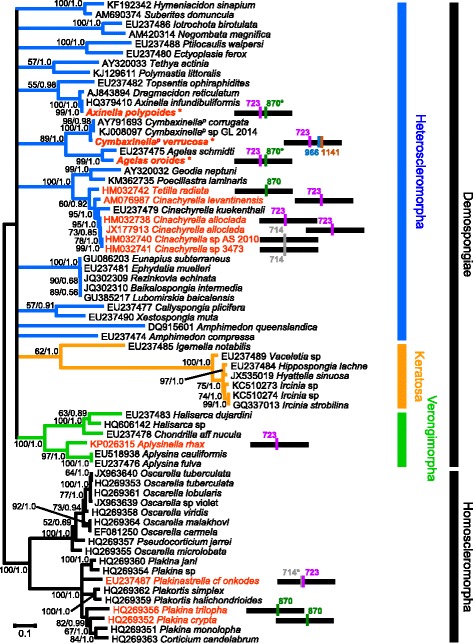
COI maximum likelihood tree. Phylogeny of Demospongidae (colored branches) and Homoscleromorpha (black branches) based on 1,566 bp from the COI gene. Species possessing an intron in their COI CDS are marked in red. All other sequences are proven to be intron free except for AJ843894 *Dragmacidon reticulatum*, HQ379410 *Axinella infundibuliformis* and KJ008097 *Cymbaxinella* ^*p*^ sp.GL 2014, for which only the barcoding region has been sequenced. The horizontal black lines represent a schematic of the COI CDS and the colored vertical bars with numbers denote intron locations. Introns with degenerated homing endonuclease are indicated with a star. Sequences obtained in this work are indicated in bold and starred; the remaining sequences were downloaded from NCBI. ML bootstrap support/Bayesian posterior probabilities are indicated near the corresponding nodes. Branches with a ML bootstrap value below 50 % were collapsed

To further rule out the possibility that the detected introns reflect contaminations from non-sponge taxa, we reconstructed the phylogenetic tree of the COI CDS, including representatives of demosponge and homoscleromorph major clades [22]. The topology obtained (Fig. 1) agrees with the accepted view of sponge phylogeny [22]. In the reconstructed tree, the sequenced COI sponge fragments were nested within their expected genera, suggesting that the COI sequences are genuine sponge sequences and not contaminations. Notably, sponges closely related to the sponges from which the COI fragments were sequenced, lacked introns, thus supporting the view that these introns were horizontally transferred (see below).

### Group I Introns 723 and 870

**Fig. 2:**
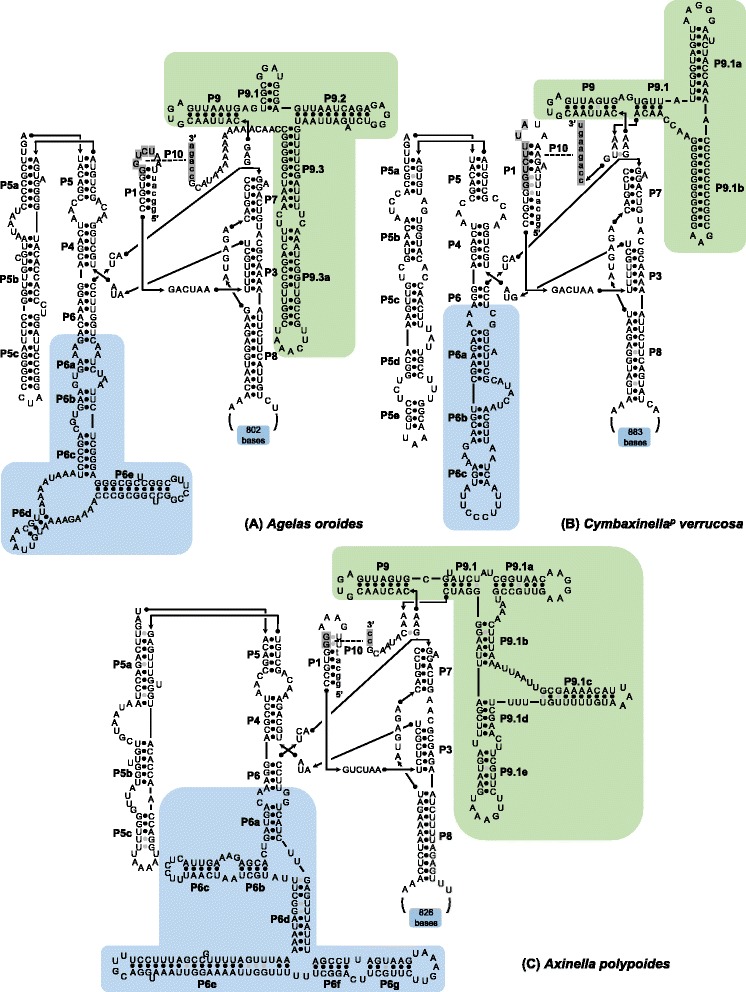
Predicted secondary structure of *A. oroides*, *A. polypoides* and *C. verrucosa* introns 723 (Group I). The exon and intron bases are indicated in lower-case and upper-case letters, respectively. The conserved sequences (P, Q, R, S) of the intron core and the base-paired regions P1–P10 follow the standard Group I intron scheme [47]. Regions with substantial structural differences among species are indicated with a colored background

**Fig. 3:**
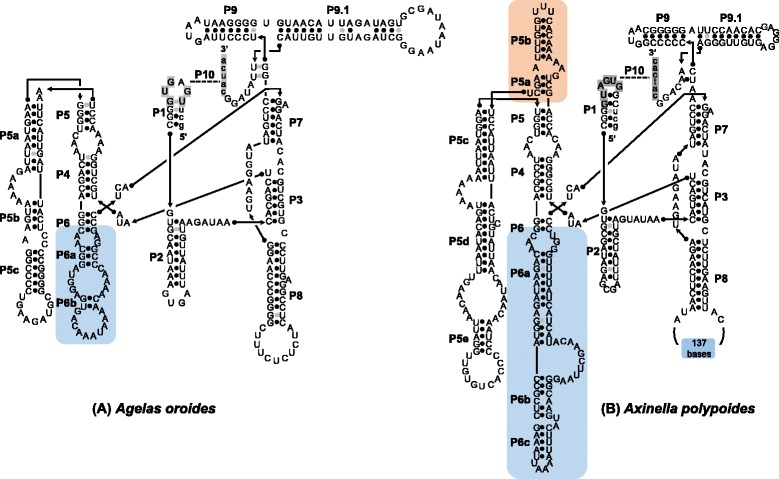
Predicted secondary structure of *A. oroides* and *A. polypoides* introns 870 (Group I). The exon and intron bases are indicated in lower-case and upper-case letters, respectively. The conserved sequences (P, Q, R, S) of the intron core and the base-paired regions P1–P10 follow the standard Group I intron scheme [47]. Regions with substantial structural differences among species are indicated with a colored background

The core structures of Group I introns 723 and 870, present in the three sequenced COI genes, were identified by the CITRON program [23]. Their predicted secondary structures follow the general structure of introns 723 and 870 as previously described by Szitenberg et al. [13]. Specifically, introns 723 are characterized by the absence of a P2 region, a rather large P6 region and a complex structure of the P9 region (Fig. 2). Introns 870 are, in contrast, characterized by the presence of a P2 region (Fig. 3). However, despite these global structure similarities to Tetillidae and Plakinidae introns, the introns 723 and 870 of *A. oroides*, *C. verrucosa* and *A. polypoides* differ in their P5, P6, and P9 structures from previously identified introns. As a case in point, the P5 of the *A. polypoides* intron 870 possesses an additional stem (indicated in orange in Fig. 3). Similarly, the P9 of the *A. oroides* intron 723 possesses an additional stem when compared to *C. verrucosa* and Tetillidae, while the P9 of the same intron in *A. polypoides* possesses two additional stems. Finally, the P6 of *C. verrucosa* intron 723 lacks stems d and e when compared to *A. oroides* and Tetillidae, while the P6 of *A. polypoides* possesses two additional stems.

Blastp 2.2.32+ [24] searches (http://blast.ncbi.nlm.nih.gov/) against the non-redundant protein database of the National Center for Biotechnology Information (NCBI) (accessed May 2015), revealed that all three introns 723 include a LAGLIDADG ORF of 948-1,176 bp, which is mainly encoded in the loop of the P8 element. However, no significant similarity (i.e., with an e-value <1E-05) to known proteins was found when conducting Blastp searches using the ORF present in introns 870 of *A. oroides* and *A. polypoides* as query. The ORFs detected in the P8 element of introns 870 of these two species were shorter (255 and 426 bp, respectively) than the LAGLIDADG ORF of other sponge introns 870 (888 bp in *Tetilla radiata*). A DNA alignment of the ORFs and LAGLIDADG CDSs identified in sponge introns 870 revealed the presence of numerous deletions and frameshift mutations in *A. oroides* and *A. polypoides* ORFs (Additional file 2). These two highly degenerated LAGLIDADG ORFs were thus not included in the phylogenetic reconstruction of the LAGLIDAG relationships.

### LAGLIDADG phylogeny

**Fig. 4:**
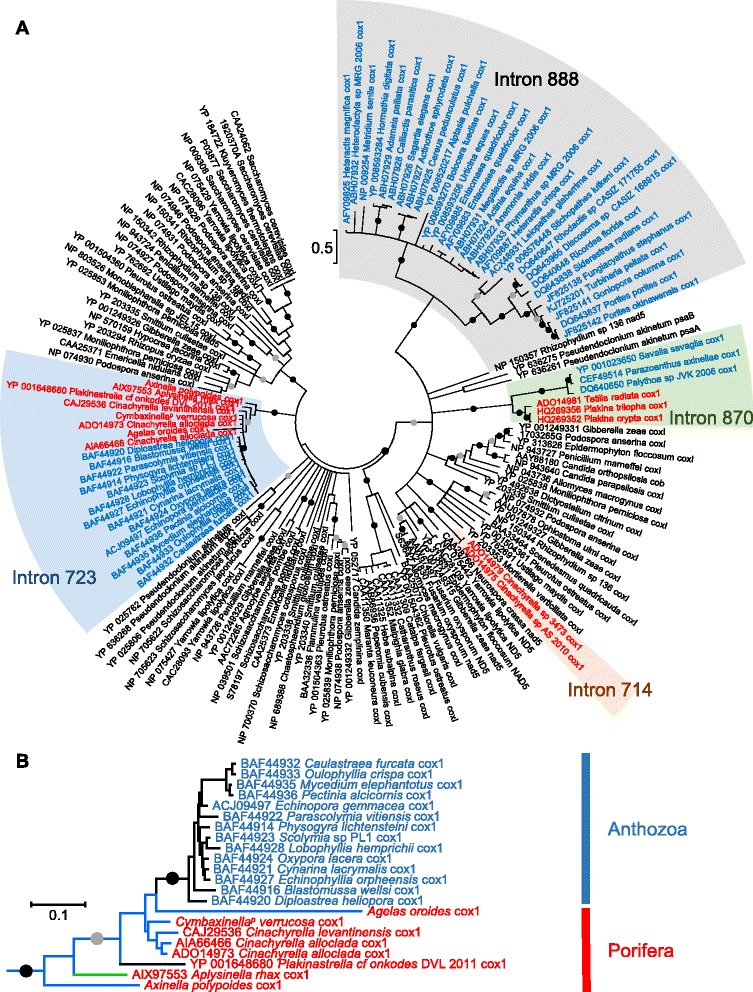
LAGLIDADG phylogenetic relationships. Sponge and cnidarian sequence names are in red and blue, respectively. Solid circles indicate branches with high support values (ML BP ≥ 95 and Bayesian PP = 1.0); grey circles indicate branches with moderate support values (95 > ML BP ≥ 75 and Bayesian PP ≥ 0.90). **a** Maximum likelihood tree. LAGLIDADG clades from introns 714, 723, 870 and 888 are indicated with red background, blue background, green background and grey background, respectively. Branches with a ML bootstrap value below 50 % were collapsed. **b** Bayesian relationships among intron 723 sequences. Branch colors follow the branch colors of Fig. 1. Specifically, Homoscleromorpha and Anthozoa are indicated by black branches, while Heteroscleromorpha and Verongimorpha are indicated by blue and green branches, respectively

To identify the origin of the putative LAGLIDADG ORFs encoded within introns 723, the new sequences were added to the LAGLIDADG dataset of Szitenberg et al. [13], together with closely related sequences available in NCBI (see Methods). In the reconstructed phylogenetic tree (Fig. 4a), the new putative LAGLIDADG sequences cluster with other metazoan LAGLIDADG sequences encoded within introns 723. The monophyly of intron 723 LAGLIDADGs is given maximal support (BP = 100, PP = 1.0). The relationships among the metazoan LAGLIDADGs, however, do not match the species tree (e.g., Fig. 1). Indeed, within the intron 723 clade, the cnidarian sequences appear to be nested among sponge sequences (see also [15]). Specifically, the Tetillidae, *A. oroides* and *C. verrucosa* sequences appear to be closer to the cnidarian sequences, while the *A. polypoides* sequence is the first diverging sequence of the clade (Fig. 4b).

### Group II introns 966 and 1141

**Fig. 5:**
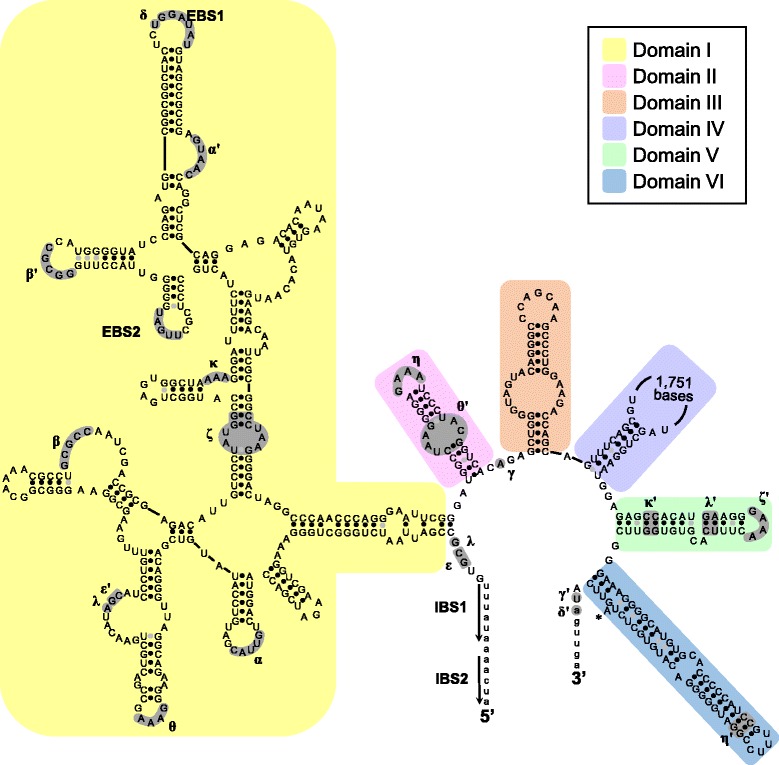
Predicted secondary structure of *C. verrucosa* intron 966 (Group II). The exon and intron bases are indicated in lower-case and upper-case letters, respectively. The conserved domains (I-VI) follow the standard Group II introns scheme [3, 25, 26]. Amino-acids involved in conserved tertiary base pairing and tetraloops are shaded

**Fig. 6:**
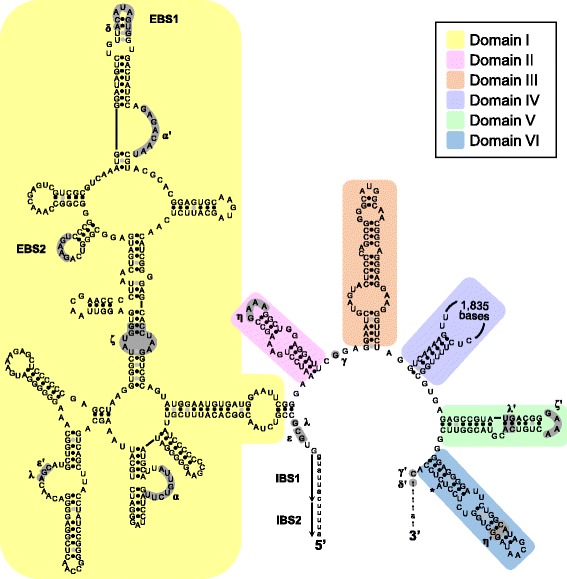
Predicted secondary structure of *C. verrucosa* intron 1141 (Group II). The exon and intron bases are indicated in lower-case and in upper-case letters, respectively. The conserved domains (I-VI) follow the standard Group II introns scheme [3, 25, 26]. Amino-acids involved in conserved tertiary base pairing and tetraloops are shaded

The introns 966 and 1141 present in the *C. verrucosa* sequence were characterised as Group II introns (see Methods). Specifically, their putative secondary structure could be organized into six stem-loop domains radiating from a central core (Figs. 5 and 6) following the model of Michel et al. [25] and Toor et al. [26]. The introns start and end with the consensus sequences GUGYG and YAY respectively. The Watson-Crick base-pairing regions and interacting elements EBS1/IBS1, EBS2/IBS2, α/α’, γ/γ’, δ/δ’, ε/ε’, λ/λ’ and the tetraloops η/η’, ζ/ζ’ could be recognised in both introns. However, the base-pairing region β-β’ and tetraloops θ- θ’ κ- κ’ elements, whose absence has been noted in other Group II introns [27], could only be identified in intron 966. The secondary structures thus indicate that both *C. verrucosa* introns belong to Group IIA1 [28]. Blastp 2.2.32+ [24] searches (http://blast.ncbi.nlm.nih.gov/) against the non-redundant protein database of NCBI (accessed May 2015) indicated that both introns 966 and 1141 encode a putative RT ORF (of 2199-2220 bp), which is mainly encoded in domain IV.

### RT phylogeny

**Fig. 7:**
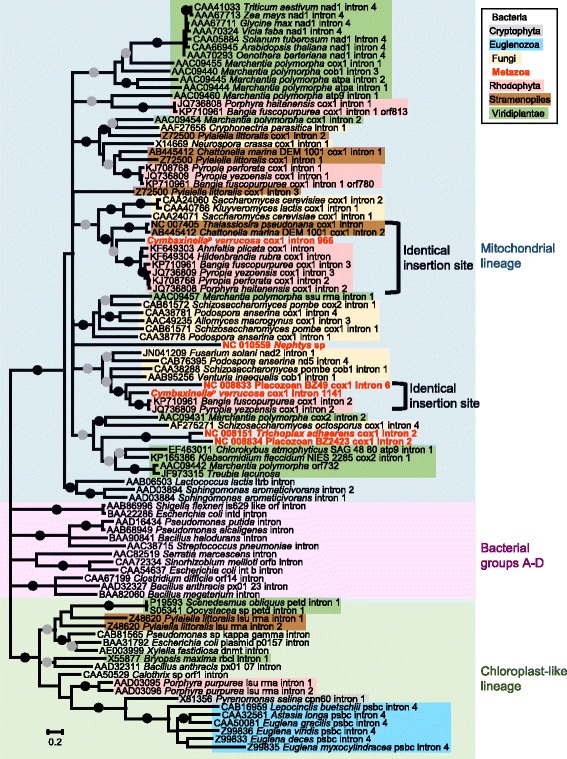
Reverse transcriptase maximum likelihood tree. Following Zimmerly et al. [29], the tree was divided into three regions: mitochondrial lineage, bacterial groups and chloroplast-like lineage. Solid circles indicate branches with high support values (ML BP ≥ 95 and Bayesian PP = 1.0); grey circles indicate branches with moderate support values (95 > ML BP ≥ 75 and Bayesian PP ≥ 0.90). Branches with a ML bootstrap value below 50 % were collapsed

To identify the origin of the putative RT ORFs encoded within introns 966 and 1141, the new RT sequences of *C. verrucosa* were added to the RT dataset of Zimmerly et al. [29], together with closely-related sequences and metazoan mitochondrial RT sequences available in NCBI (see Methods). The phylogenetic tree (Fig. 7) shows that all metazoan RT sequences belong to the mitochondrial lineage. However, the metazoan RTs do not form a monophyletic clade among mitochondrial sequences. Rather, they belong to four distinct clades. One of these clades is comprised of the sequence of *C. verrucosa* intron 1141, which clusters together with Rhodophyta and Stramenopiles sequences. Interestingly, a careful inspection of all introns within this clade indicated that they are all inserted at orthologous positions in the COI sequence, although for several sequences, the intron insertion site is incorrectly annotated in NCBI. The RT sequence of *C. verrucosa* intron 966 forms another clade with the RT sequence of Placozoan BZ49 COI intron 6 and Rhodophyta sequences. Again, we verified that all species within this clade share the same intron insertion site in their COI sequence. Two additional metazoan branches consist of: 1- the *Nephtys* RT sequence; and 2- the NC008151 *Trichoplax adhaerens* COI intron 2 RT and the NC008834 Placozoan BZ2423 COI intron 2 RT. These last two sequences appear to be related to Viridiplantae sequences.

## Discussion

Our results show that introns in sponges are not limited to the families Tetillidae, Aplysinidae, and Plakinidae, but are also present in Agelasidae, Axinellidae, and Hymerhabdiidae. We also show that not only Group I introns but also Group II introns are present in sponges. The new data allow us to discuss the putative origin of the mitochondrial introns.

Introns can be transmitted either horizontally or vertically. The results presented here reinforce the idea that both Group I and Group II mitochondrial introns are horizontally transmitted in sponges [12, 13, 15], as observed, for example, in the mitochondrial genome of fungi [8, 30, 31], sea anemones [11, 18], or plants [32]. First, the distribution of each sponge intron appears to be extremely patchy among animals, which suggests numerous independent losses for each intron. Additionally, if the introns were vertically transferred, we would expect each animal intron clade to follow the species phylogeny, while in fact both the LAGLIDADG and the RT trees (Figs. 4 and 6) contradict the species tree (Fig. 1).

In agreement with the phylogenetic inferences, the intron secondary structure are often more similar between distantly related sponges than closely related ones. For example, the introns 870 of *Tetilla radiata* (Tetillidae, Demospongiae), *Plakina crypta* and *Plakina trilopha* (Plakinidae, Homoscleromorpha) are structurally more similar to one another than to the introns 870 of *A. polypoides* and *A. oroides* ([13], Fig. 3)*.* Similarly, the difference in secondary structure among the sponge introns 723 suggests independent insertion events in Tetillidae, Axinellidae and Agelasida.

Interestingly, not all introns were found to encode a functional HEG. Goddard and Burt proposed a cyclical model of intron gain and loss [8], which explains the existence of degenerate HEG. In this model, later called “intron life cycle” or “HEG life cycle” (e.g., [5, 18]), sequences with intron free insertion site are repeatedly invaded by mobile introns with a functional HEG. Following fixation of the intron at the insertion site, the HEG degenerates and becomes non-functional. Over time, not only the HEG but the entire intron is lost, thus returning to the initial intron-free state from which a new cycle of intron gain and loss can start again.

Emblem et al. [11], distinguished five stages in the cycle of invasion of mitochondrial Group I introns: 1 - young introns have an HEG ORF in frame with the upstream COI exon; 2 - mature introns express free-standing HEG; 3 - old introns show degenerate HEG ORF; or, 4 - no HEG at all; and 5 - the introns are lost. According to this model, introns 723 of *C. verrucosa* and *A. polypoides* appear to be in stage 1; intron 723 of *A. oroides* is in stage 2; while introns 870 of *A. polypoides* and *A. oroides* are in stage 3 and 4, respectively.

Introns 723 of the Agelasida *A. oroides* and *C. verrucosa* are in different stages of their intron life cycle and show very different secondary structures and rates of evolution, as evident from the difference in branch length in the LAGLIDADG tree (Fig. 4) when compared to the COI tree (Fig. 1). It is thus likely that different horizontal gene transfers independently occurred at the same insertion site within these two Agelasida species. It is interesting to note that introns 870 are found in different stages across sponges. They are in stage 1 in *Tetilla radiata*, *Plakina crypta* and *Plakina trilopha* and in stages 3 and 4 in *A. polypoides* and *A. oroides*, further supporting the horizontal transfer hypothesis. Introns 723, on the contrary, are only found in either stage 1 or 2 in sponges and corals, suggesting a recent infection of these two lineages.

Although many new complete mitochondrial genomes have been sequenced since the discovery of the first sponge intron by Rot et al. [12], the origins of the sponge Group I introns are still unclear. Indeed, in the LAGLIDADG phylogenetic analysis, the sponge sequences do not have any evident sister clade taxa, except for anthozoan cnidarians (Fig. 4). However, because the anthozoan sequences appear nested within sponge sequences it is more probable that a similar fungal or algal donor was responsible for both the sponge and the anthozoan transfers (see [10]). The donor of the two Group II introns found in *C. verrucosa,* however, was probably a red alga, since in both cases the RT sequence of *C. verrucosa* was related to red alga RTs. Additionally, for both Group II introns, the *C. verrucosa* and the Rhodophyta introns had an orthologous insertion site in the COI gene. Nonetheless, because the grouping of the *C. verrucosa* and Rhodophyta sequences is not strongly supported, we cannot exclude the possibility that the introns were transmitted by another donor. Indeed, the diatom *Thalassiosira pseudonana* and the raphidophyte *Chatonella marina* share closely-related RT sequences and an orthologous insertion site with the RT encoded in intron 966. Similarly, the Placozoa BZ49 shares a closely-related RT sequence and an orthologous insertion site with the *C. verrucosa* intron 1141. The fact that all the above-mentioned species share the same mitochondrial genetic code may also facilitate the transfer of introns among these distant eukaryote lineages. Sequencing of additional mitochondrial genomes from marine eukaryotes is expected to better resolve the origin of the animal mitochondrial introns.

It has been suggested that budding ability might favour the transmission of introns since in budding species the transmission of genetic material is not limited to the germline [13]. Specifically, it was noted that both Plakinidae and Tetillidae possess budding abilities [13]. Interestingly, external budding and asexual reproduction have also been described in members of the genera *Cymbaxinella*^*p*^ [33] and *Agelas* [34], supporting the view that budding might explain why introns are more frequent in some sponge families than in others. The evolutionary rate is another parameter that has been suggested to influence intron transmission. Indeed, in fast evolving species, the intron splicing site, whose sequence conservation is essential, has less chance to remain intact, thus impeding the insertion of novel introns [35, 36]. This might explain why no introns have been found in the mitochondrial genome of the faster evolving medusozoan cnidarian, such as hydroids, jellyfish, and box jellies [37], even though they, like all cnidarians, possess the possibility to reproduce asexually.

## Conclusion

The huge increase in sponge sequence data, leads to novel insights regarding the evolution of the mitochondrial genomes of sponges (e.g., [16, 38, 39]). Here, we provide novel data and analyses that show that sponge mitochondrial introns encompass both Group I and Group II introns. While the origin of Group I introns is still uncertain, our results show that the Group II introns of *C. verrucosa* are related to those in Rhodophyta. However, we cannot exclude the possibility that the donor might be a lineage whose sequences are not yet available in public databases. Our analyses further indicate that sponge introns are present in more sponge families than previously thought. Notably, all sponge introns discovered hitherto are either inserted within or after the reverse primer used for barcoding. Our small survey using a different reverse primer suggests that up to one in ten of sponge species could possess an intron. In addition, while writing this manuscript, we have been informed of the discovery of a novel Group I intron in the sponge *Stupenda singularis* (Tetractinellida, Stupendidae) inserted at position 387 in COI [40]. Since for most taxa the barcoding region is the only mitochondrial region that has been sequenced, it is possible that mitochondrial intron diversity in sponges, and in bilaterian animals in general, might be underestimated.

## Methods

### DNA extractions and amplifications

Tissue samples of *A. oroides* (voucher TAU Po.25598), *C. verrucosa* (voucher TAU Po.25600) and *A. polypoides* (voucher TAU Po.25597) preserved in 95 % ethanol, were obtained from the Steinhard Museum of Natural History (Additional file 3). Since the samples were provided by the museum no ethical approval was required. The specimens, which belong to species that are neither protected nor endangered, were collected as part of a survey of the sponge diversity along the Mediterranean coast of Israel (permits 40165, 40171, 40741, 40744; the Israel Nature and National Park Protection Authority). *A. oroides* and *C. verrucosa*, genomic DNA was extracted following the procedure of Fulton et al. [41]. *A. polypoides* was extracted with the Qiagen DNAeasy kit #69504 following the manufacturer’s instructions.

Amplification of the COI gene was performed in two steps. Detailed information concerning the PCR conditions are outlined in Additional file 4. For all three samples a first amplification was performed with the primers Cox_Calc_D1 (5'-TWTNTTCWACHAAYCAYAAAGAYAT-3′) and Cox_Calc_R1 (5′-AARAARTGTTGRGGGAARAADGT-3′) [13]. Because the amount of amplified DNA was insufficient for direct sequencing after this first amplification step, re-amplifications were performed. For *A. polypoides* and *A. oroides*, re-amplification using 1 microliter of the first PCR product as template was performed with the primers LCO1490 (5′-GGTCAACAAATCATAAAGATATTGG-3′) [42] and Cox1R1 (5′-TGTTGRGGGAAAAARGTTAAATT-3′) [12].

For *C. verrucosa* the primer Cox_Calc_R1 showed non-specific annealing to position ~600 bp of the COI sequence. The complete sequence of *C. verrucosa* was therefore obtained in three fragments. First, a shorter fragment embracing the 5′ region of the COI fragment was obtained by performing a re-amplification of the Cox_Calc_D1-Cox_Calc_R1 product with the primers LCO1490 and COX550PoeR (5′- CATAGTWATMGCCCCNGCTAATAC-3′**)**. Based on the sequence of the Cox1D1-Cox550PoeR fragment and the sequence of *A. polypoides*, a forward primer, CoxAxiD2, was designed (5′-ATWAGATTAGAATTATCKGCTCC-3′). A middle fragment was then obtained by performing a re-amplification of the Cox_Calc_D1-Cox_Calc_R1 fragment with the CoxAxiD2-Cox1R1 primers. New primers Verrucosa_D1 (5′- GTTATTGTCTCTGCCAGTTTTAGCCGGG-3′) and Averr2014Da (5′-TGCCAGAAGTTTACATTTTAATATTGCC-3′) were then designed based on this middle region. The 3′ end of the sequence (including the Group II introns) was obtained with a different set of amplification. The first PCR was performed applying only the forward primer Verrucosa_D1 on a *C. verrucosa* genomic extract. This procedure was aimed to enrich the template in mitochondrial copies. A re-amplification was then performed using Averr2014Da and Cox_Calc_R1.

Sequencing of the COI genes was performed using primers specifically designed for each sample. In the case of *A. oroides,* Sequencing of the COI gene was complicated by the presence of a secondary structure. To facilitate sequencing, PCR fragments of *A. oroides* were cloned into the pJET #K1231 vector (Thermo Scientific). The new sequences were deposited in the EMBL-EBI European Nucleotide Archive under accession numbers LN868208- LN868210.

### Intron position and secondary structures

The position of each intron was determined by performing a manual alignment of the sequence obtained with the complete COI sequence of closely-related species. Following Szitenberg et al. [13] introns were named based on insertion position with respect to the COI sequence of *Amphimedon queenslandica* complete COI. Specifically, in *A. oroides* and *A. polypoides* sequences, two introns were inserted after positions 723 and 870, when using the sequence of *Amphimedon queenslandica* as reference. Similarly, three introns were identified in the sequence of *C. verrucosa,* inserted after positions 723, 966 and 1141, also when using the sequence of *A. queenslandica* as reference.

The secondary structure of Group I introns was determined following Rot et al. [12]. First, the CITRON program [23] was used to identify the core structure of the Group I introns (i.e., P3, P4, P7, P8). The Mfold web server (http://unafold.rna.albany.edu/?q=mfold) [43] was then used to identify the peripheral hairpin structures (i.e., P1, P2, P5, P6, P9). The secondary structure of the Group II introns was determined manually following the instructions given in the Database for bacterial Group II introns [44], and using the structure model of subgroup IIA described in Michel et al. [25] and Toor et al. [26]. The Mfold web server [43] was used to confirm stem folding. Blastp 2.2.32+ searches [24] were conducted with default settings against the non-redundant protein database of NCBI (accessed May 2015) to identify the protein family to which the ORF protein encoded within each intron belonged.

### Phylogenetic reconstruction based on COI CDS

To confirm the phylogenetic position of the obtained COI sequences all complete mitochondrial genome sequences of demosponges and homoscleromorphs available in NCBI (accessed March 2015) were downloaded, and the complete COI CDSs were extracted from these sequences. All sponge COI CDSs known to harbour an intron [12–15] were also added to this dataset. Unfortunately, the COI sequence of *S. singularis* was not yet available in NCBI while preparing this manuscript and could not be included in our phylogenetic analyses. Since few complete mitochondrial genomes were available from species closely-related to *A. polypoides* and *C. verrucosa*, Blastn 2.2.32+ searches [24] were conducted with default settings against the non-redundant protein database of NCBI (accessed March 2015) to identify similar sequences. The closest hits, sequences AJ843894 *Dragmacidon reticulatum,* HQ379410 *Axinella infundibuliformis* (for *A. polypoides*) and KJ008097 *Cymbaxinella*^*p*^ sp.GL 2014 (for *C. verrucosa*) were thus added to the dataset.

A translation alignment was performed on the CDSs using the L-INS-I algorithm of MAFFT v7.017 [45] as implemented in Geneious 6.1.8 (www.geneious.com). Positions with more than 50 % of missing data were excluded from the alignment. The final alignment is available as supplementary material (Additional file 5). The COI alignment includes 66 species and 1,566 characters of which 700 are constant and 808 are parsimony informative.

Phylogenetic trees were reconstructed under the Maximum likelihood (ML) and Bayesian criteria. ML trees were built with RAxML version 8.0.26. The dataset was partitioned based on codon positions and each position was assumed to evolve under an independent GTR + Gamma model of sequence evolution. The ML tree was identified using 100 starting trees. Support values were computed using 100 slow bootstrap replicates. The Bayesian tree was reconstructed with MrBayes v3.2.2 using the same partitions and model as in the ML analysis. Two independent runs, each with four chains, were sampled every 500 generations. Each chain was run for 100,000,000 generations. Posterior probabilities (PP) were computed after excluding the first 25 % of the trees (burnin). Convergence of the run was assessed by verifying that the average standard deviation was below 0.01 before the burnin threshold, and that the potential scale reduction factors of the parameters were equal to 1. The homoscleromoph sequences were used as root for the demosponge tree and *vice versa*.

### Phylogenetic reconstruction based on LAGLIDADG protein sequences

To reconstruct the phylogenetic origin of the LAGLIDADG ORF encoded within Group I introns, the dataset of Szitenberg et al. [13] was downloaded, along with additional mitochondrial LAGLIDADG sequences of anthozoan and sponges available in NCBI. Specifically, tBlastn 2.2.32+ searches [24] were conducted with default settings against the non-redundant nucleotide database of NCBI (accessed May 2015) using different LAGLIDADG as query (i.e., a representative of each metazoan LAGLIDADG clade was considered) in order to identify closely related unannotated LAGLIDAG sequences that were not included in [13]. Blastp 2.2.32+ and tBlastx 2.2.32+ searches [24] were then conducted with default settings against the non-redundant protein database of NCBI (accessed May 2015) to identify non-metazoan sequences sister to the anthozoan and sponge LAGLIDADG. No sequences were included at this stage.

LAGLIDADG sequences were aligned using MAFFT v7.017 under the L-ins-I algorithm. Positions with more than 50 % of missing data were excluded from the alignment. The alignment is available as supplementary material (Additional file 6). The LAGLIDADG alignment includes 93 species and 595 characters of which 2 are constant and 592 are parsimony informative.

Phylogenetic tree reconstructions were performed using RaxML 8.0.26 and MrBayes v3.2.2. The best model of evolution was found to be the VT + I + G + F using ProtTest 3.4 [46]. Because RaxML does not recommend modelling among site-rate variation using both the gamma parameter and the invariant parameter, sequences were analysed under the VT + Gamma + F model. The ML tree was identified using 100 starting trees and support values were computed using 100 slow bootstrap replicates. The MrBayes analysis was also performed under the VT + Gamma + F model. Two independent runs, each with four chains, were sampled every 500 generations. Each chain was run for 70,000,000 generations. Posterior probabilities (PP) were computed after excluding the first 25 % of the trees. Because the average standard deviation of split frequencies never dropped below 0.018, which indicates adequate but not very good convergence, the analysis was run a second time under the same conditions. Similar topologies with almost identical posterior probabilities were obtained in the two independent Bayesian analyses, indicating that convergence was reached. The rooting of the tree followed Szitenberg et al. [13].

### Phylogenetic reconstruction based on RT protein sequences

To reconstruct the phylogenetic origin of the RT ORF encoded within the Group II introns of *C. verrucosa*, the approach of Vallès et al. [17] was followed. Specifically, the dataset of Zimmerly et al. [29] was downloaded, and mitochondrial RT sequences of metazoans (i.e., *Nephtys, Trichoplax* and *C. verrucosa*) were added. Blastx 2.2.32+ and Blastp 2.2.32+ searches were then conducted with default settings against the non-redundant protein database of NCBI (accessed May 2015) to identify sequences with an E-value = 0.0 and at least 45 % identity to the metazoan RTs. These sequences were added to the dataset. RT sequences were aligned with MAFFT v7.017 under the L-ins-I algorithm. Positions with more than 50 % missing data were excluded from the alignment. The alignment is available as supplementary material (Additional file 7). The RT alignment includes 145 species and 291 characters of which one is constant and 288 are parsimony informative.

Phylogenetic tree reconstructions were performed using RaxML 8.0.26 and MrBayes v3.2.2 as described above for the LAGLIDADG analysis. The best model of evolution was also found to be VT + I + G + F using ProtTest 3.4 [46]. The only difference was that MrBayes chains were run for 38,000,000 generations. Convergence of the run was assessed as described for the COI analysis. The rooting of the tree followed Zimmerly et al. [29].

## Availability of supporting data

Sequences were deposited in the EMBL-EBI European Nucleotide Archive under accession numbers LN868208- LN868210. Sequence alignments used in the phylogenetic analysis are included as (Additional files 5, 6 and 7).

## Additional files

Additional file 1:
**List of species sequenced as part of the sponge survey.** (XLSX 10 kb) Additional file 2:
**Alignment of ORF 870.** DNA sequence alignment, in Fasta format, of the ORF 870 of *Tetilla radiata*, *Plakina trilopha, Plakina crypta, Agelas oroides* and *Axinella polypoides*. (FASTA 5 kb) Additional file 3:
**Sample origin.** (XLSX 11 kb) Additional file 4:
**PCR conditions.** (DOCX 13 kb) Additional file 5:
**COI CDS alignment.** DNA sequence alignment, in Nexus format, used to reconstruct the phylogenetic tree presented in Fig. 1. (NEX 144 kb) Additional file 6:
**LAGLIDADG protein alignment.** Protein sequence alignment, in Nexus format, used to reconstruct the phylogenetic tree presented in Fig. 4. (NEX 213 kb) Additional file 7:
**Reverse transcriptase protein alignment.** Protein sequence alignment, in Nexus format, used to reconstruct the phylogenetic tree presented in Fig. 7. (NEX 182 kb)

## References

[1] Lang BF, Laforest MJ, Burger G (2007). Mitochondrial introns: a critical view. Trends Genet.

[2] Medina M, Collins AG, Takaoka TL, Kuehl JV, Boore JL (2006). Naked corals: skeleton loss in Scleractinia. Proc Natl Acad Sci U S A.

[3] Lambowitz AM, Zimmerly S (2011). Group II introns: mobile ribozymes that invade DNA. Cold Spring Harbor Perspect Biol.

[4] Zimmerly S, Semper C (2015). Evolution of group II introns. Mob DNA.

[5] Haugen P, Simon DM, Bhattacharya D (2005). The natural history of group I introns. Trends Genet.

[6] Dellaporta SL, Xu A, Sagasser S, Jakob W, Moreno MA, Buss LW (2006). Mitochondrial genome of *Trichoplax adhaerens* supports Placozoa as the basal lower metazoan phylum. Proc Natl Acad Sci U S A.

[7] Signorovitch AY, Buss LW, Dellaporta SL (2007). Comparative genomics of large mitochondria in placozoans. Plos Genetics.

[8] Goddard MR, Burt A (1999). Recurrent invasion and extinction of a selfish gene. Proc Natl Acad Sci U S A.

[9] Brugler MR, Opresko DM, France SC (2013). The evolutionary history of the order Antipatharia (Cnidaria: Anthozoa: Hexacorallia) as inferred from mitochondrial and nuclear DNA: implications for black coral taxonomy and systematics. Zool J Linn Soc.

[10] Fukami H, Chen CA, Chiou CY, Knowlton N (2007). Novel group I introns encoding a putative homing endonuclease in the mitochondrial *cox1* gene of Scleractinian corals. J Mol Evol.

[11] Emblem Å, Okkenhaug S, Weiss ES, Denver DR, Karlsen BO, Moum T (2014). Sea anemones possess dynamic mitogenome structures. Mol Phylogenet Evol.

[12] Rot C, Goldfarb I, Ilan M, Huchon D (2006). Putative cross-kingdom horizontal gene transfer in sponge (Porifera) mitochondria. BMC Evol Biol.

[13] Szitenberg A, Rot C, Ilan M, Huchon D (2010). Diversity of sponge mitochondrial introns revealed by *cox 1* sequences of Tetillidae. BMC Evol Biol.

[14] Gazave E, Lapébie P, Renard E, Vacelet J, Rocher C, Ereskovsky AV (2010). Molecular phylogeny restores the supra-generic subdivision of homoscleromorph sponges (Porifera, Homoscleromorpha). PLoS One.

[15] Erpenbeck D, Aryasari R, Hooper JN, Wörheide G (2015). A mitochondrial intron in a verongid sponge. J Mol Evol.

[16] Wang XJ, Lavrov DV (2008). Seventeen new complete mtDNA sequences reveal extensive mitochondrial genome evolution within the Demospongiae. PLoS One.

[17] Vallès Y, Halanych KM, Boore JL (2008). Group II introns break new boundaries: presence in a bilaterian’s genome. PLoS One.

[18] Goddard MR, Leigh J, Roger AJ, Pemberton AJ (2006). Invasion and persistence of a selfish gene in the Cnidaria. PLoS One.

[19] Gazave E, Carteron S, Chenuil A, Richelle-Maurer E, Boury-Esnault N, Borchiellini C (2010). Polyphyly of the genus *Axinella* and of the family Axinellidae (Porifera: Demospongiae^p^). Mol Phylogenet Evol.

[20] Morrow C, Cárdenas P (2015). Proposal for a revised classification of the Demospongiae (Porifera). Front Zool.

[21] Morrow CC, Picton BE, Erpenbeck D, Boury-Esnault N, Maggs CA, Allcock AL (2012). Congruence between nuclear and mitochondrial genes in Demospongiae: a new hypothesis for relationships within the G4 clade (Porifera: Demospongiae). Mol Phylogenet Evol.

[22] Lavrov DV, Wang XJ, Kelly M (2008). Reconstructing ordinal relationships in the Demospongiae using mitochondrial genomic data. Mol Phylogenet Evol.

[23] Lisacek F, Diaz Y, Michel F (1994). Automatic identification of group-I intron cores in genomic DNA-sequences. J Mol Biol.

[24] Altschul SF, Madden TL, Schaffer AA, Zhang J, Zhang Z, Miller W (1997). Gapped BLAST and PSI-BLAST: a new generation of protein database search programs. Nucleic Acids Res.

[25] Michel F, Umesono K, Ozeki H (1989). Comparative and functional anatomy of group II catalytic introns - a review. Gene.

[26] Toor N, Hausner G, Zimmerly S (2001). Coevolution of group II intron RNA structures with their intron-encoded reverse transcriptases. RNA.

[27] Kamikawa R, Masuda I, Demura M, Oyama K, Yoshimatsu S, Kawachi M (2009). Mitochondrial Group II Introns in the Raphidophycean Flagellate *Chattonella* spp. suggest a Diatom-to-*Chattonella* Lateral Group II Intron Transfer. Protist.

[28] Dai L, Toor N, Olson R, Keeping A, Zimmerly S (2003). Database for mobile group II introns. Nucleic Acids Res.

[29] Zimmerly S, Hausner G, Wu X-C (2001). Phylogenetic relationships among group II intron ORFs. Nucleic Acids Res.

[30] Mardanov AV, Beletsky AV, Kadnikov VV, Ignatov AN, Ravin NV (2014). The 203 kbp mitochondrial genome of the phytopathogenic fungus *Sclerotinia borealis* reveals multiple invasions of introns and genomic duplications. PLoS One.

[31] Férandon C, Moukha S, Callac P, Benedetto J-P, Castroviejo M, Barroso G (2010). The *Agaricus bisporus cox1* gene: the longest mitochondrial gene and the largest reservoir of mitochondrial group I introns. PLoS One.

[32] Vaughn JC, Mason MT, Sperwhitis GL, Kuhlman P, Palmer JD (1995). Fungal origin by horizontal transfer of a plant mitochondrial group I intron in the chimeric *cox**I* gene of *Peperomia*. J Mol Evol.

[33] Boury-Esnault N (1970). Un phénomène de bourgeonnement externe chez l’éponge *Axinella damicornis* (Esper). Cah Biol Mar.

[34] Hoppe WF (1988). Reproductive patterns in three species of large coral reef sponges. Coral Reefs.

[35] Lynch M, Koskella B, Schaack S (2006). Mutation pressure and the evolution of organelle genomic architecture. Science.

[36] Swithers KS, Senejani AG, Fournier GP, Gogarten JP (2009). Conservation of intron and intein insertion sites: implications for life histories of parasitic genetic elements. BMC Evol Biol.

[37] Kayal E, Roure B, Philippe H, Collins A, Lavrov D (2013). Cnidarian phylogenetic relationships as revealed by mitogenomics. BMC Evol Biol.

[38] Lavrov DV, Pett W, Voigt O, Wörheide G, Forget L, Lang BF (2013). Mitochondrial DNA of *Clathrina clathrus* (Calcarea, Calcinea): six linear chromosomes, fragmented rRNAs, tRNA editing, and a novel genetic code. Mol Biol Evol.

[39] Erpenbeck D, Voigt O, Worheide G, Lavrov DV (2009). The mitochondrial genomes of sponges provide evidence for multiple invasions by Repetitive Hairpin-forming Elements (RHE). BMC Genomics.

[40] Kelly M, Cárdenas P. An unprecedented new genus and family of Tetractinellida (Porifera, Demospongiae) from New Zealand’s Colville Ridge, with a new type of mitochondrial group I intron. *Zool* J Linn Soc. In press

[41] Fulton TM, Chunwongse J, Tanksley SD (1995). Microprep protocol for extraction of DNA from tomato and other herbaceous plants. Plant Mol Biol Rep.

[42] Folmer O, Black M, Hoeh W, Lutz R, Vrijenhoek R (1994). DNA primers for amplification of mitochondrial cytochrome *c* oxidase subunit I from diverse metazoan invertebrates. Mol Mar Biol Biotechnol.

[43] Zuker M (2003). Mfold web server for nucleic acid folding and hybridization prediction. Nucleic Acids Res.

[44] Candales MA, Duong A, Hood KS, Li T, Neufeld RAE, Sun R (2012). Database for bacterial group II introns. Nucleic Acids Res.

[45] Katoh K, Standley DM (2013). MAFFT multiple sequence alignment software version 7: improvements in performance and usability. Mol Biol Evol.

[46] Darriba D, Taboada GL, Doallo R, Posada D (2011). ProtTest 3: fast selection of best-fit models of protein evolution. Bioinformatics.

[47] Cech TR, Damberger SH, Gutell RR (1994). Representation of the secondary and tertiary structure of group I introns. Nat Struct Biol.

