# Supplementary material for: Mitochondrial group I and group II introns in the sponge orders Agelasida and Axinellida
Source: BMC Evol Biol. 2015 Dec 12;15:278. doi: 10.1186/s12862-015-0556-1 (PMC4676843; doi:10.1186/s12862-015-0556-1)
Supplement: Additional file 4: — PCR conditions. (DOCX 13 kb) [file 12862_2015_556_MOESM4_ESM.docx]

**Detailed PCR conditions**

**General reaction mixture for PCR (total 25 μl)**

| DNA (50-10 ng/μl) | 1 μl |
| --- | --- |
| 10X Ex Taq Buffer | 2.5 μl |
| dNTPs (2.5 mM each) | 1.5 μl |
| Direct primer (5μM) | 1.5 μl |
| Reverse primer (5μM) | 1.5 μl |
| TaKaRa Ex Taq (5 units/μl) | 0.2 μl |
| Sterilized distilled water | 16.8 μl |

PCR mixtures only differ in the primers used (see text).

**PCR conditions**

1. Denaturation cycle

94°C for 3 min

1. 5 cycles with

94°C for 30 sec

55°C for 30 sec

72°C for 1 min 30 sec for short fragments (600-1,200 kb), 2-4 min for

longer fragments (up 7kbp)

1. 35 cycles with

94°C for 30 sec

50°C for 30 sec

72°C for 1 min 30 sec for short fragments (600-1,200 kb), 2-4 min for

longer fragments (up 7kbp)

1. Final elongation

72°C for 10 min
